# CD59a deficiency exacerbates influenza-induced lung inflammation through complement-dependent and-independent mechanisms

**DOI:** 10.1002/eji.200636755

**Published:** 2007-05

**Authors:** M Paula Longhi, Anwen Williams, Matthew Wise, B Paul Morgan, Awen Gallimore

**Affiliations:** 1 Department of Medical Biochemistry and Immunology, School of Medicine, Cardiff UniversityCardiff, UK; 2 Department of Rheumatology, School of Medicine, Cardiff UniversityCardiff, UK; 3 Adult Critical Care, University Hospital WalesCardiff, UK

**Keywords:** Inflammation, Lung, T cells, Virus

## Abstract

Influenza-specific immune activity not only promotes virus clearance but also causes immunopathology, thereby underlining the importance of mounting a measured anti-viral immune response. Since complement bridges both the innate and adaptive immune systems and has been implicated in defence against influenza, the role of the complement regulator CD59a in modulating the response to influenza was explored. For this purpose, immune responses to influenza virus, strain E61-13-H17, in mice deficient in the complement regulator protein CD59a (Cd59a^–/–^ mice) were compared to those in wild-type mice. The severity of lung inflammation was significantly enhanced in the lungs of Cd59a^–/–^ mice with increased numbers of infiltrating neutrophils and CD4^+^ T cells. When complement was inhibited using soluble complement receptor1, the frequency of lung-infiltrating neutrophils in influenza-infected Cd59a^–/–^ mice was much reduced whilst numbers of CD4^+^ T cells remained unchanged. These results demonstrate that CD59a, previously defined as a complement regulator, modulates both the innate and adaptive immune response to influenza virus by both complement-dependent and-independent mechanisms.

## Introduction

Influenza virus infection represents a major health problem, causing high morbidity and mortality worldwide. Studies of mouse models have revealed that the pathology resulting from influenza infection reflects both direct virus-induced damage as well as indirect effects of the immune response stimulated following infection. Cells of the innate and adaptive immune system have been implicated in virus-induced lung pathology and the extent of cellular infiltration, including neutrophils, eosinophils and T cells, has been shown to correlate with the degree of lung damage [1–5]. These cells migrate to the lungs, leading to disruption and occlusion of airways [3, 5, 6]. Release of pro-inflammatory cytokines and chemokines by both infected and immune cells further promotes influenza-induced pathology through direct effects on lung tissue and by further recruitment of inflammatory cells and T cells to the site of infection [7–10]. The involvement of the immune system in lung pathology is also corroborated by histological studies of lung biopsies of humans with influenza pneumonia, which revealed the presence of lymphocytic infiltrates in the injured tissue [11].

The complement system bridges both innate and adaptive immunity and promotes migration of immune cells into lungs. Administration of cobra venom factor, a potent activator of the complement system, has been used to induce lung injury in a mouse model. Complement activation products, generated as consequence of systemic complement activation by cobra venom factor, caused neutrophil migration and sequestration in lungs [12, 13]. Moreover, complement is known to influence both virus-specific immune responses in the lung and lung injury [14–18]. In particular, mice deficient in complement component C3, the lynchpin of the complement cascade, display reduced influenza virus-specific T cell responses in infected lungs, implying that complement activation plays an important role in T cell activation and/or recruitment [12]. Collectively, these studies imply that activation of the complement system markedly influences the extent of cellular infiltration into the lungs of influenza-infected mice and, as a consequence, the degree of lung damage.

We reasoned that defects in complement regulation would also influence the clinical course of influenza infection by permitting enhanced complement activation. In order to test this hypothesis, wild-type (WT) mice and mice lacking CD59a, the membrane regulator of complement membrane attack complex (MAC) formation, were infected with influenza virus. A comparison of the degree of cellular infiltration into the lungs of both mouse groups revealed an enhanced infiltration of neutrophils, CD4^+^ T cells and CD4/CD8 double-positive (DP) cells in the lungs of the Cd59a^–/–^ mice. This was accompanied by an increase in lung pathology, observed by histology, in the mice lacking Cd59a. Thus, our results demonstrate that clinical disease following influenza infection is enhanced in the absence of CD59a, and that severity correlated with the infiltration of neutrophils and CD4^+^ T cells into the lungs of the infected mice. Analysis of the role of complement in the enhanced injury and inflammation in the lungs of influenza-infected Cd59a^–/–^ mice revealed that increased neutrophil infiltration was dependent on complement activation whilst enhancement of the CD4^+^ T cell response was complement-independent. These results demonstrate that CD59a modulates the immune response to influenza virus by both complement-dependent and-independent mechanisms.

## Results

### Cd59a^−/−^ mice exhibit increased lung pathology compared to WT mice

**Figure 1:**
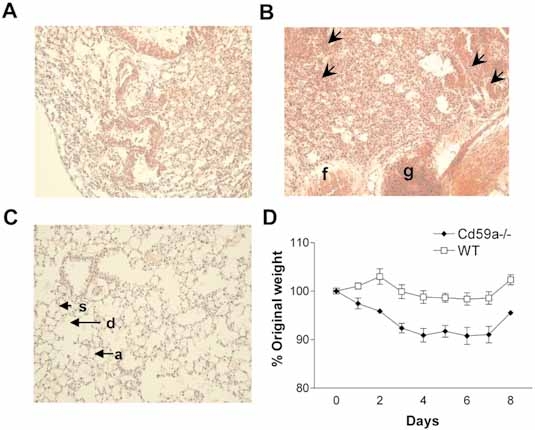
(A–C) Histological analysis of lung sections taken from Cd59a^–/–^, WT and age-matched WT controls. Lung histology (H&E stain) demonstrating pulmonary injury 8days after infection with influenza virus (*n*=9/group). Representative views of mouse lungs (×20original magnification) from WT(A) or Cd59a^–/–^ (B) mice infected with influenza are shown. A representative section from an uninfected WT control (C) is also included. In Cd59a^–/–^ mice there is mild hyperplasia of alveolar type-II cells and an extensive infiltrate of mononuclear cells into the interstitium (arrows). The alveolar walls contained dilated capillaries filled with RBC, with mild fibrotic changes (f) and perivascular lymphocytic infiltrates (g). In the healthy lung (C), the alveoli (a), alveolar septa (s) and alveolar duct (d) are marked for comparison. (D) Mice on the Balb/c background were infected with influenza virus and weight was monitored daily. Results represent mean values ± SEM of five mice per group. Statistical significance was evaluated using the Student's *t*-test. *p*value was <0.02 at all time points measured after infection.

Experimental influenza infection results in significant lung injury that can be detected by histological study of infected lungs. Cd59a^–/–^ and WT mice were infected with influenza virus (H17, H3N2) [19] intranasally (i.n.) and lungs were evaluated 8days post-infection by analysing H&E-stained sections of influenza-infected lungs. Stringent scoring of histological sections was performed by two independent investigators blinded to the various treatment regimes and mice used. Influenza-infected WT mice showed a moderate increase in leukocyte infiltration compared to uninfected mice (representative sections in Fig. 1]A–C). There was no significant difference in lung pathology between naive WT and Cd59a^–/–^ mice. In contrast, Cd59a^–/–^ mice showed pronounced and significant elevation in all parameters indicative of pulmonary injury resulting from influenza infection, namely, the degree of haemorrhage, interstitial leukocyte infiltration and perivascular lymphoid aggregation (Table 1]). Interestingly, mild fibrosis was also noted in the Cd59a^–/–^ mice but was absent in all WT mice (data not shown)

**Table 1:** Histological Analyses of Influenza-Infected Lungs

	Haemorrhagea)	Leukocyte infiltrationa)	Perivascular lymphoid aggregatesa)	Fibrosisa)	Histological scoreb)
**WT**					
1	2.5±0.5	2.5±0.5	0	0	5±1
2	2.0±1.0	2.5±0.5	0	0	4.5±2.5
3	1.5±1.5	2.5±0.5	0	0	4±2
4	0.5±0.5	3.0±1.0	0	0	3.5±1.5
5	1.5±1.5	2.5±0.5	0	0	4±2
6	0	2±0	0	0	2±0
7	1.5±1.5	1.5±1.5	0	0	3±3
8	0	0	0	0	0
9	0.5±0.5	0	0	0	0.5±0.5
					
**Mean±sem**c)	1.1±0.3	1.8±0.3	0	0	2.9±0.6
CD59^–/–^					
1	1.0±1.0	2.5±0.5	0	0	3.5±0.5
2	3.0±0	5.0±0	2.0±0	0	10±0
3	3.0±0	5.0±0	2.5±0.5	0.5±0.5	11.5±0.5
4	3.0±0	4.0±1.0	1.5±1.5	0	8.5±2.5
5	3.0±0	5.0±0	3.0±0	0.5±0.5	11.5±0.5
6	3.0±0	4.0±0	0	0	7±0
7	3.5±0.5	4.0±0	2.0±2.0	0.5±0.5	10±3
8	3.0±0	3.5±1.5	1.0±0	0	7.5±1.5
					
**Mean±sem**^c)^	2.8±0.2**	4.0±0.3*	1.5±0.4**	0.2±0.1	8.6±0.8*
					
Naive					
**WT**	0	0	0	0	0
CD59^–/–^	0	0	0	0	0
					

a) The severity of these parameters have been graded by two blinded observers

b) Cumulative histological scores for each parameter for each CD59^–/–^ and WT are presented.

c) The sum of scores for these pathological indices comprised the histological score for each section (mean±sem). There was no evidence of pathological change in either naïve CD59^–/–^ and WT mice, each having a histological score of 0, respectively. No necrosis or granuloma was observed in any of the sections assessed. * p<0.001 and ** p<0.005 (Mann Whitney U test).

Despite clear histological evidence of increased lung pathology in Cd59a^–/–^ compared to WT mice, no obvious physical symptoms of influenza infection were observed in either group. Since Balb/c mice are more likely to develop clinical symptoms than C57BL/6 (B6) mice following infection with the same dose of influenza virus (T.Hussell, personal communication), we also investigated the effect of CD59a on the outcome of influenza virus infection in Balb/c mice. For this purpose, Balb/c WT and Balb/c.Cd59a^–/–^ mice (the latter kindly provided by Professor Marina Botto, Imperial College, London, UK) were infected with the same low dose of influenza virus used to infect the B6 mice, and weight loss was monitored daily. Infection resulted in rapid weight loss in Balb/c.Cd59a^–/–^ mice while body weight in Balb/c.WT mice remained almost constant (Fig. 1D).

### Enhanced neutrophil and CD4^+^ T cell lung infiltration in infected Cd59a^–/–^ mice

**Figure 2:**
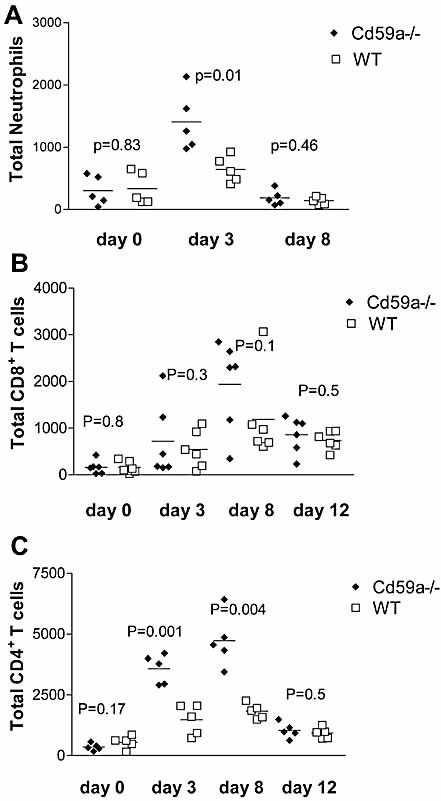
Increased immune cell recruitment in the lungs of influenza-infected Cd59a^–/–^ mice. Cd59a^–/–^ and WT mice (*n*=5/group) were infected i.n. with influenza virus. Lungs were harvested at the times indicated and lung-infiltrating neutrophils (A) CD8^+^ (B) and CD4^+^ T cells (C) were enumerated by flow cytometry. Neutrophils were identified as SSC^high^ CD11b^+^ Gr1^high^ F4/80^–^. Each symbol represents an individual mouse. Means are represented in all graphs. Statistical significance was evaluated using Student's *t*-test.

In order to compare cellular infiltrates in the lungs of WT and Cd59a^–/–^ mice on the B6 background, lungs were harvested 3and 8days post-infection with influenza virus, and the different cell types analysed by flow cytometry. No difference was observed in the extent of NK cell, macrophage and eosinophil recruitment between the groups of mice (data not shown), but higher numbers of neutrophils were observed in the lungs of Cd59a^–/–^ compared to WT mice (Fig. 2A). Influenza infection of Balb/c.Cd59a^–/–^ mice replicated the previous findings with B6.Cd59a^–/–^ mice in that the degree of cellular infiltration was enhanced in mice deficient for CD59 compared to their WT counterpart (data not shown).

Total numbers of lung-infiltrating lymphocytes were analysed by flow cytometry at day3, 8 and12 post-infection. Both Cd59a^–/–^ and WT B6 mice showed a peak of T cell infiltration at day8 post-infection that decreased significantly by day12 (Fig. 2B,C). No significant difference was observed in numbers of lung-infiltrating CD8^+^ T cells between the groups of mice (Fig. 2B). The number of CD4^+^ T cells recovered from the lungs at day3 post-infection was strikingly higher in Cd59a^–/–^ compared to WT mice (Fig. 2C). Similar results were observed at day8 post-infection and by day12, numbers of CD4^+^ T cells in lungs were similar for both groups of mice. Similar results were obtained using Balb/c.Cd59a^–/–^ mice when compared to the relevant controls (data not shown). The total numbers of infiltrating cells in Balb/c.Cd59a^–/–^ mice were greater than in B6.Cd59a^–/–^ mice, correlating with the more pronounced clinical symptoms observed in this strain.

### CD4/CD8 double-positive cells in the lungs of infected mice

**Figure 3:**
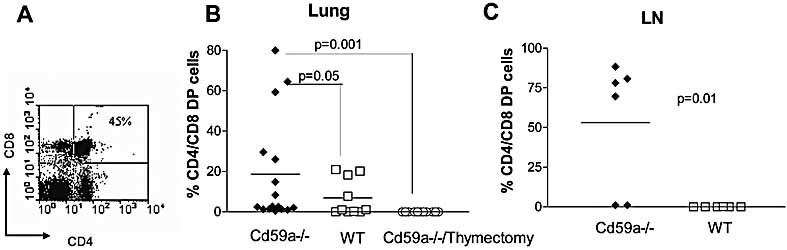
Increased frequency of CD4/CD8 DP cells in Cd59a^–/–^ mice. Mice were infected with influenza virus and the number of infiltrating CD4/CD8 DP cells analysed. A representative dot plot of CD4/CD8 expression is shown in (A). Three days post-infection, lungs (B) and LN (C) were harvested and analysed for the presence of CD4/CD8 DP cells enumerated by flow cytometry. Cd59a^–/–^ mice were thymectomized and, after 2wk, mice were infected with influenza virus. Presence of CD4/CD8 DP cells was analysed in lungs 3days after infection (B). Non-thymectomized mice were used as controls. Each symbol represents an individual mouse (*n*=6/group). Means are indicated in all graphs. Statistical significance was evaluated by Student's *t*-test.

Phenotypic analyses of the cells infiltrating the lungs of influenza-infected mice revealed a large population of CD4/CD8 DP lymphocytes (Fig. 3A). These cells were analysed by two-colour flow cytometry at day3 and8 post-infection. At 3days post-infection (Fig. 3B), CD4/CD8 DP cells were observed in the lungs of both WT and Cd59a^–/–^ B6 mice but significantly higher percentages were observed in Cd59a^–/–^ mice. No DP cells were observed in either group by day8 post-infection (data not shown). The percentages of the DP cells varied between 1% and almost 80% in Cd59a^–/–^ mice but did not exceed 40% in WT mice. CD4/CD8 DP cells were also found in the draining lymph nodes of Cd59a^–/–^ mice but not in the draining lymph nodes of WT mice (Fig. 3C).

CD4/CD8 DP cells are present in the thymus where they differentiate into either CD4 or CD8 single-positive cells before emigrating into the periphery. By staining with a panel of antibodies, we found that the DP cells in the lungs and lymph nodes of influenza-infected mice resembled those found in the thymus (TCR^low^ CD4^intermediate^ CD8^high^ CD25^low^ CD62L^low^) (data not shown).

To explore the possibility that the DP cells were immature thymocytes that had emigrated prematurely from the thymus in response to virus infection, mice were thymectomized, infected 2wk later with influenza virus and lungs harvested 3days post-infection. The experiment was performed in Cd59a^–/–^ mice due to the higher number of CD4/CD8 DP cells recruited in lungs after influenza infection. Whilst CD4/CD8 DP cells were found in 70% of control Cd59a^–/–^ mice, none were found in any of the thymectomized mice, confirming a thymic origin for the DP cells (Fig. 3B). The functional significance of these DP cells remains unclear since we found that the cells were unable to produce IL-4 or IFN-γ after stimulation with PMA and ionomycin (data not shown). The DP cells may not participate in the anti-influenza immune response and their presence in the lung may simply be a consequence of an inflammatory process occurring in the vicinity of the thymus.

### Enhanced influenza-specific CD4^+^ T cell activity in Cd59a^–/–^ mice

**Figure 4:**
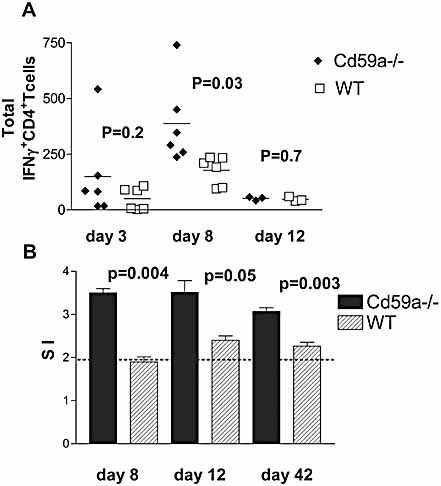
Cd59a^–/–^ mice exhibited increased CD4^+^ T cell activity following influenza infection. (A) Cd59a^–/–^ and WT mice (*n*=6/group) were infected i.n. with influenza virus. Lungs were harvested and homogenates were stimulated with PMA/ionomycin for 4h. CD4^+^ IFN-γ-producing T cells were enumerated by flow cytometry. Each symbol represents an individual mouse. Means are represented in all graphs. (B) After 8, 12and 42days, CD4^+^ T cells were purified from splenocytes and stimulated with APC loaded with UV-inactivated virus. Influenza-specific proliferation was detected by [^3^H]thymidine incorporation at day6. Stimulation index (SI) was calculated by dividing specific cpm by the background. Mice were analysed individually and values shown are the mean ± SEM (*n*=3/group). The results are representative of two independent experiments. Statistical significance was evaluated using Student's *t*-test.

As shown in Fig. 2C, an increased frequency of single-positive CD4^+^ T cells was observed in the lungs of influenza-infected Cd59a^–/–^ compared to WT B6 mice. In order to study the function of these cells, intracellular cytokine staining was performed to assess IFN-γ production by the lung-infiltrating CD4^+^ T cells in Cd59a^–/–^ and WT mice. IFN-γ-producing cells were mainly detected at day8 post-infection and the number was significantly higher in Cd59a^–/–^ compared to WT mice (Fig. 4A). To determine whether the increase in the number of CD4^+^ T cells in the lungs of Cd59a^–/–^ mice was due to an increase in the activity of antigen-specific T cells, influenza-specific CD4^+^ T cell proliferation was examined. Cd59a^–/–^ and WT mice were infected with influenza virus, and CD4^+^ T cells purified from spleens at day8, 12 and42 after infection. Influenza-specific proliferation of CD4^+^ T cells was significantly increased in Cd59a^–/–^ compared to WT mice at all time points tested (Fig. 4).

### Role of complement in enhanced lung infiltration in Cd59a^–/–^ mice

**Figure 5:**
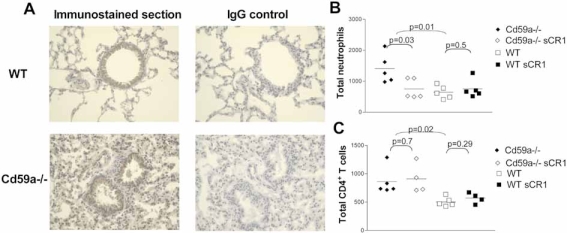
Enhanced influenza-induced lung inflammation in Cd59a^–/–^ mice is mediated by complement-dependent and-independent mechanisms. (A) MAC deposition was analysed by C9 staining on lung sections. Representative views of mouse lungs (×40original magnification) from WT (top) or Cd59a^–/–^ (bottom) mice infected with influenza are shown. Arrowheads indicate lung airway epithelium staining. (B,C) Complement was inhibited *in vivo* by administration of sCR1 (i.v.) daily and mice were infected with influenza virus. After 3days, lungs were harvested and total numbers of lung-infiltrating neutrophils (B) and CD4^+^ T cells (C) were determined by flow cytometry. Each symbol represents an individual mouse (*n*=5/group). Means are represented in all graphs. Statistical significance was evaluated using Student's *t*-test.

CD59a expression was previously described in the alveoli and the bronchial epithelium in mouse lungs [20. The absence of CD59a in the Cd59a^–/–^ mice has been associated with increased complement activation and MAC deposition in several diseases [21, 22]. Staining for MAC in B6 mice infected with influenza virus revealed a more extensive deposition of MAC along the respiratory airways of CD59a^–/–^ compared to WT mice (Fig. 5A). In order to assess the effect of complement activation on cellular infiltration into the lungs of WT and Cd59a^–/–^ mice, complement was inhibited by daily injection of sCR1 (i.v.) for the first 3days of influenza infection as previously described [23. After 3days, lungs were harvested and total numbers of infiltrating cells analysed by flow cytometry.

No change in numbers of CD8^+^ T cells, NK cells, macrophages or eosinophils was observed in Cd59a^–/–^ mice after sCR1 administration, whereas the number of neutrophils infiltrating in the lungs of these mice was significantly reduced and was not different from levels found in infected WT mice after complement inhibition (Fig. 5B). In contrast, numbers of infiltrating CD4^+^ T cells remained unchanged after sCR1 administration, indicating that modulation of the CD4^+^ T cell response by CD59a in experimental influenza infection is complement-independent (Fig. 5C).

## Discussion

The immune response to influenza virus represents a double-edged sword; mounting an adequate response is crucial for virus clearance, but over-exuberant recruitment of immune cells into the lung can result in tissue damage [6, 24]. Indeed, the extent of the innate and adaptive immune response to influenza virus infection in experimental mouse models correlates with the extent of lung injury, weight loss and mortality. Complement bridges both the innate and adaptive immune systems, is known to cause activation and migration of leukocytes, and has been implicated in defence against influenza [14]. Roles of complement regulators in modulating the response to influenza have not previously been explored.

Here we analysed whether the complement regulator CD59a influences the immune response to influenza virus. Histological studies of lung sections revealed increased lung injury in influenza-infected mice lacking CD59a. This was accompanied by an enhanced infiltration of neutrophils and lymphocytes into the lungs of infected mice that may contribute to lung injury through direct cytotoxicity and/or the release of immune mediators that further amplify the inflammatory infiltrate [12, 25, 26]. A parallel study in Balb/c mice indicated that Balb/c mice lacking CD59a showed increased disease, monitored by cellular infiltration of the lungs and weight loss following infection, when compared with WT Balb/c controls.

In order to establish the role of complement, we treated Cd59a^–/–^ and WT mice with the soluble complement inhibitor sCR1 over the course of infection. Treatment with sCR1 did not significantly alter influenza-induced neutrophil infiltration in WT mice but markedly reduced neutrophil influx in Cd59a^–/–^ mice, reaching levels similar to those found in WT mice. These findings show that the augmented neutrophil response in Cd59a^–/–^ mice was due to enhanced complement activation. Absence of CD59a, by rendering cells more susceptible to MAC-mediated damage or killing, might indirectly cause enhanced local complement activation and generation of complement activation products such as the anaphylatoxins C3a and C5a. Both C3a and C5a have been shown previously to act as chemoattractants for neutrophils and may be responsible for directing the migration of neutrophils into influenza-infected lungs ([27], reviewed in [28]). In addition, it has been demonstrated that MAC formation can indirectly influence the recruitment of inflammatory cells. For example, deposition of sub-lytic amounts of MAC on human endothelial cells *in vitro*promotes production of IL-8 and MCP-1, chemokines for neutrophils and monocytes, respectively [29, 30]. Inhibition of complement had no effect on neutrophil numbers in WT mice, probably due to the efficient natural regulation of complement activation in these mice.

Higher numbers of lung-infiltrating CD4^+^ T cells were observed in influenza virus-infected Cd59a^–/–^ mice compared to WT mice on days3 and8 post-infection, but by day12 this difference no longer existed. CD4^+^ T cells from both groups of mice produced equal amounts of IFN-γ on a per cell basis, but the greatly increased numbers of lung-infiltrating CD4^+^ T cells in Cd59a^–/–^ mice resulted in higher total levels of the cytokine, which is known to have direct anti-viral effects, at the site of virus replication. Mice were infected with low doses of the H17 virus and virus levels were undetectable in Cd59a^–/–^ and WT mice at day8, a time point described as the peak of infection [31, 32], indicating that both groups of mice efficiently controlled the virus.

Since it has previously been reported that complement activation promotes the activity of influenza-specific T cells in the lungs, it was possible that the effect of CD59a on T cell activity was an indirect effect attributable to increased complement activation [14, 33, 34]. However, inhibition of complement activity using sCR1 did not alter the enhancement of CD4^+^ T cell responses observed in the Cd59a^–/–^ mice, indicating that the effect of CD59a deficiency on CD4^+^ T cell activity was complement-independent. These data support our previous finding from studies of vaccinia virus infection that CD59a expression down-modulates the activity of anti-viral CD4^+^ T cells in a complement-independent manner [23, 35]. The precise mechanism through which CD59a down-modulates T cell activity is not yet known but evidence suggests that a negative signal is delivered to the T cell as a result of CD59a binding to an as yet unidentified ligand on APC [23, 35].

Analysis of cells infiltrating the lungs of influenza-infected WT and Cd59a^–/–^ mice revealed a population of CD4/CD8 DP cells appearing early in the course of infection. Higher numbers were observed in the lungs of Cd59a^–/–^ mice compared to WT mice, and DP cells were found in the draining lymph nodes only in Cd59a^–/–^ mice. Phenotypic examination indicated that these cells resembled immature DP T cells isolated from thymus. Thymic origin was confirmed by showing that DP cells were not found in mice thymectomised prior to influenza infection. CD4/CD8 DP cells have been found previously in mice infected with influenza virus in the NIH/S mouse strain. These cells were found in the lung within days of infection, and were CD4^intermediate^ CD8^high^ DP cells that did not express TCR and did not produce IFN-γ upon stimulation [36]. These findings correlate with our findings and suggest that these cells play no role in anti-viral immunity and do not impinge upon the course of infection or pathology. The increased incidence of DP cells in influenza-infected Cd59a^–/–^ mice compared to WT mice is most likely a consequence of the more profound inflammatory response to influenza virus observed in these mice.

Overall, the results of experiments described in this study indicate that influenza-induced immunopathology is exacerbated in mice lacking CD59a. Increased pathology correlated with higher numbers of lung-infiltrating neutrophils and CD4^+^ T cells. Whilst a robust immune response is clearly critical for virus clearance and survival of the host, it is also important that the immune system is kept in check since over-exuberant immune responses can result in deleterious effects on the host leading to increased morbidity and mortality. Our findings identify the complement regulator CD59a as a regulator of the host response to influenza infection both by impinging on complement-driven responses and by directly down-modulating antiviral T cells. If human CD59 is shown to have a similar role in modulating human CD4^+^ T cells it is possible that targeted use of recombinant CD59 may be useful for limiting immunopathology at sites of inflammation.

## Materials and methods

### Mice

B6 (H-2^b^) mice (WT) were obtained from Harlan (Oxford, UK). B6.129-Cd59a^tm1Bpm^ (Cd59a^–/–^) mice were generated as previously described [37] and back-crossed onto the B6 background for eight generations. Cd59a^–/–^ mice back-crossed eight generations onto the Balb/c background were kindly provided by Professor Marina Botto (Imperial College, London, UK). Mice used in the experiments were approximately 6wk old and bred in isolators. During experimental procedures mice were housed in Scantainers. All experiments were performed in compliance with UK Home Office regulations.

### Antibodies and fluorescence staining

Anti-CD4-PerCPCy5.5, anti-CD8-Cy5 and anti-F4/80-Cy5 mAb were purchased from Caltag Laboratories (Burlingame, CA). Anti-IFN-γ-FITC, anti-CD25-PE, anti-Gr1-PerCPCy5.5, anti-CD11c-APC, anti-CD11b-FITC, anti-NK1.1-PE and anti-B220-FITC mAb were purchased from BD PharMingen (San Diego, CA). Directly conjugated antibodies were utilized for cell surface staining. Cells were incubated with 0.5µg/mL of antibody for 30min before washing and re-suspension in FACS buffer (PBS + 2% FCS + 2mM EDTA). Intracellular cytokine staining was performed by incubating lung-derived lymphocytes for 4h at 37°C in the presence of ionomycin (1µg/mL), PMA (20ng/mL) and monensin (3µM) (Sigma-Aldrich, St. Louis, MO). Staining was performed according to the manufacturer's instructions (BD PharMingen). In all cases, cells were re-suspended in FACS buffer and analysed by flow cytometry (FACSCalibur®; Beckton Dickinson, Mountain View, CA).

### Infection with influenza virus and determination of anti-virus response

Recombinant influenzaA virus strain E61-13-H17 (H17, H3N2) amplified in embryonated chicken eggs, was obtained from the National Institute for Medical Research (London, UK). The virus was titrated from allontoic fluid by performing a haemagglutination assay as previously described [38]. Mice were infected i.n. with 20haemagglutination units of Influenza virus H17 in 20µL of PBS. Seroconversion was confirmed by ELISA as described previously [38]. At day3 and8 after infection, mice were sacrificed and lungs perfused with PBS. Lungs were harvested for virus titres, histology and immunostaining, and spleens harvested for CTL and CD4^+^ T cell proliferation assays (day8 post-infection). For cell staining purposes, single-cell suspensions were prepared from the lung tissue by mechanical disaggregation. In order to measure memory anti-virus responses, spleens were harvested approximately 6wk after infection; then CTL and CD4^+^ T cell proliferation assays were performed. *In vivo* complement inhibition (>90% inhibition compared to control mice during the experiment) was achieved by daily i.v. injections of mice with 20mg/kg of sCR1 (gift of TCell Sciences) as previously described [23]. Cell infiltration in lungs was analysed 3days post-infection.

### Influenza virus-specific CD4^+^ T cell proliferation

CD4^+^ T cells from single-cell suspensions of splenocytes were purified by positive MACS MicroBead selection (Miltenyi Biotec, Bergisch Gladbach, Germany) according to the manufacturer's instructions. Influenza-specific CD4^+^ T cell proliferation assays were performed 8and 42days after infection by incubating 10^5^CD4^+^ T cells with 6×10^5^irradiated splenocytes previously incubated with UV-inactivated virus. Cell proliferation was assessed by thymidine incorporation or CFSE FACS analysis at day6.

### Histology

Lungs were perfused with PBS and fixed in Zinc fixative (0.1M Tris-HCl pH7.4 with 0.05% Ca-acetate, 0.5% Zn-acetate and 0.5% Zn-chloride) [39]. The lungs were then embedded in paraffin wax, sections (5µM) were cut and stained with hematoxylin and then counterstained with eosin (H&E). The sections were graded subjectively using various parameters indicative of pulmonary inflammation, namely evidence of haemorrhage (0–3), the degree of interstitial leukocyte infiltration (0–5), the extent of perivascular lymphoid aggregate formation (0–4) and the presence of fibrotic lesions (0–1). The sum of the scores for each parameter comprised the histological score (0–13) for each animal. Two investigators, who were blinded to the identity of each histological specimen, scored each section.

For MAC deposition, lung paraffin sections were first treated with H_2_O_2_ to eliminate endogenous peroxidase activity. Sections were subsequently treated with 10% normal swine serum to reduce non-specific staining. Sections were then incubated with rabbit anti-rat C9 IgG (manufactured in our laboratory and cross-reactive with mouse C9 [40]) or control rabbit IgG followed by biotinylated swine anti-rabbit Ig. Antibody labelling was detected using a high-sensitivity streptavidin-horseradish peroxidase conjugate and diaminobenzidine as chromogen (Vector Laboratories, Burlingame, CA).

## Abbreviations

B6: C57BL/6
DP: double-positive
MAC: membrane attack complex
